# Clinical and genetic analysis of *ERCC8*-Related cockayne syndrome: hepatic dysfunction as a biomarker, anhidrosis as a rare feature, and rehabilitation outcomes for ankle contractures

**DOI:** 10.3389/fgene.2025.1591551

**Published:** 2025-08-06

**Authors:** Jing Chen, Wei Su, Dan Gao, Fangfang Liu, Shuang Chen, Wenhan Zhang, Min Peng, Tao Lei, Hongmin Zhu

**Affiliations:** ^1^ Wuhan Children’s Hospital, Tongji Medical College, Huazhong University of Science and Technology, Wuhan, China; ^2^ Chigene (Beijing) Translational Medical Research Center Co., Ltd., Beijing, China

**Keywords:** cockayne syndrome, ERCC8 gene, hepatic dysfunction, ankle contracture, cerebral palsy

## Abstract

**Objectives:**

Cockayne syndrome (CS), a rare hereditary neurodegenerative disorder caused by pathogenic variants in *ERCC6* (CSB) and *ERCC8* (CSA), often clinically overlaps with cerebral palsy (CP), leading to misdiagnosis. This study evaluates the role of genetic testing in differential diagnosis, examines hepatic dysfunction as a biomarker of disease severity, and delineates clinical characteristics of CSA-related CS.

**Methods:**

A retrospective case series of eight CSA-related CS patients was conducted. Clinical data, neuroimaging, genetic profiles, and hepatic function were analyzed. Disease severity was classified according to established CS subtypes (I–III).

**Results:**

All patients (6 males, 2 females) presented with early-onset motor delay and spasticity, initially misdiagnosed as CP. Genetic testing identified pathogenic *ERCC8* variants, including exon deletions (Exon4; Exon6-12), a nonsense (c.856A>T), frameshift (c.394_398del), and splice-site (c.618-2A>G) variant, confirming autosomal recessive inheritance (compound heterozygous/homozygous). Subtype distribution included CS I (n = 5), CS II (n = 2), and CS III (n = 1). CS II cases exhibited earlier diagnosis and classic CS features. Hepatic dysfunction correlated with disease severity, worsening with progression. Achilles tendon contractures developed in all patients; systematic rehabilitation (n = 5) significantly reduced contracture severity compared to non-rehabilitated cases (n = 3). Two patients displayed anhidrosis, a rarely reported dermatological manifestation.

**Conclusion:**

Genetic testing is essential to differentiate CSA-related CS from CP. Hepatic dysfunction serves as a biomarker for disease progression, warranting routine monitoring. Rehabilitation therapy mitigates Achilles tendon contractures, underscoring its clinical value. This study expands the phenotypic spectrum of CSA-related CS by identifying anhidrosis as a rarely reported feature, providing insights for diagnosis and management.

## 1 Introduction

Cockayne syndrome (CS) is a rare, progressive autosomal recessive disorder marked by multisystem involvement. With an estimated incidence of 1 in 250,000 live births and a prevalence of 2.5 per million, CS is historically defined by diagnostic criteria established by Nance and Berry (1992) and classified into three subtypes: CS I (moderate), CS II (severe), and CS III (mild) (Nance and Berry, 1992; Kubota et al., 2015; Karikkineth et al., 2017). CS I typically present with normal birth growth but developmental abnormalities within the first 2 years, progressing to severe neurological dysfunction and disability, with survival into the second or third decade. CS II, the most severe form, manifests at birth with profound growth retardation, minimal neurodevelopment, and congenital anomalies such as cataracts and joint contractures, often leading to mortality within the first decade. CS III, the mildest form, features delayed symptom onset, relatively normal early growth and cognition, and slower progression, with some patients surviving for decades and pursuing formal education (Natale, 2011; Laugel, 2013; Karikkineth et al., 2017). Hallmark clinical features include progressive neurodegeneration (e.g., microcephaly, cognitive impairment, gait disturbances), cachectic dwarfism, progeroid facial appearance, photosensitivity, and pigmentary retinopathy, with a mean life expectancy of 12 years. Neuroimaging typically reveals hypomyelination, cerebral atrophy, and intracranial calcifications. Due to phenotypic heterogeneity and clinical overlap with cerebral palsy (CP), misdiagnosis is common (Vafaee et al., 2018; Pearson et al., 2019).

CS arises primarily from pathogenic variants in *ERCC6* (CSB) and *ERCC8* (CSA), which disrupt transcription-coupled nucleotide excision repair (TC-NER), impairing DNA damage response and driving multisystem pathology (Pascucci et al., 2018; Pines et al., 2018; Cordisco et al., 2019; Carnie et al., 2024). Rarely, variants in other NER genes, such as *ERCC2* (XPD), *ERCC3* (XPB), and *ERCC5* (XPG), can also cause CS (Natale and Raquer, 2017). While *ERCC6* variants account for 70%–75% of cases, *ERCC8* variants are more prevalent in Asian populations and underlie CS-A (OMIM#216400) (Liu et al., 2024). The 396-amino acid CSA protein, encoded by *ERCC8* (chr5q12.1), functions within a Cullin4 E3 ubiquitin ligase complex to mediate TC-NER and regulate transcriptional recovery post-DNA damage (Laugel et al., 2010; Vessoni et al., 2020; Duong et al., 2022). Loss-of-function variants in *ERCC8* result in developmental defects, neurological deterioration, and premature aging. Despite advances in genetic diagnostics, CSA-specific clinical features and therapeutic strategies remain poorly characterized. Hepatic dysfunction—commonly observed as elevated transaminases—has not been systematically evaluated for its correlation with disease severity. Similarly, musculoskeletal complications, such as ankle contractures, lack evidence-based management guidelines.

This retrospective study analyzed eight CSA-related CS cases to: (1) delineate clinical and genetic distinctions between CS and CP; (2) assess hepatic dysfunction as a biomarker of disease severity; and (3) evaluate rehabilitation efficacy in alleviating ankle contractures. By integrating phenotypic, genetic, and therapeutic data, this work aims to refine diagnostic precision and inform targeted interventions for CSA-related CS.

## 2 Materials and methods

### 2.1 Subjects

A retrospective study of eight pediatric patients with CSA-related Cockayne syndrome (CS) was conducted at Wuhan Children’s Hospital. Clinical data included demographic characteristics (sex, age, disease duration), clinical phenotypes (abnormal muscle tone, motor developmental delay, photosensitivity), diagnostic evaluations (neuroimaging findings such as cerebral atrophy and hypomyelination, neurophysiological studies, and hepatic function markers including serum transaminases, blood ammonia, and lipid profiles), genetic profiles derived from whole-exome sequencing (WES), and rehabilitation interventions with follow-up outcomes. The study protocol was approved by the Institutional Review Board of Wuhan Children’s Hospital (Approval No. 2022R043-E01) and adhered to the Declaration of Helsinki. Written informed consent was obtained from all participants’ legal guardians.

Diagnostic probability and disease severity were assessed using the Spitz et al. (2021) scoring system. The diagnostic likelihood score incorporated 10 clinical or 12 clinical-radiological parameters, stratifying patients into high-, moderate-, or low-probability categories. Disease severity was quantified using five clinical and developmental parameters, with higher scores indicating milder manifestations. Two senior pediatricians independently evaluated all cases, resolving discrepancies through consensus to ensure scoring consistency.

### 2.2 Genetic analysis

Whole-exome sequencing (trio-WES) was performed on probands and their parents. Peripheral blood samples were collected in EDTA anticoagulant tubes, and genomic DNA was extracted using a column-based kit (TianGen Biotech). Exome enrichment was performed using the xGen^®^ Exome Research Panel v2.0 (Integrated DNA Technologies), followed by sequencing on a DNBSEQ-T7 platform (BGI-Shenzhen). Sequencing reads were aligned to the GRCh37/hg19 reference genome using BWA, and variant calling was performed with GATK to identify single-nucleotide variants (SNVs) and small insertions/deletions (Indels). Duplicate reads were marked and removed using SAMtools and Picard, while copy number variations (CNVs) were detected via CNVnator and AMYCNE. Variants with a minor allele frequency (MAF) > 1% in population databases (1000 Genomes, gnomAD) were excluded. Missense variants were evaluated for pathogenicity using computational tools (PolyPhen-2, SIFT, REVEL), and splice-site variants were analyzed with SpliceAI and MaxEntScan. CNV annotation and ranking were performed using AnnotSV, integrating data from DGV, DECIPHER, ClinGen, and PubMed. Variants were classified according to ACMG/ClinGen guidelines as pathogenic (P), likely pathogenic (LP), variant of uncertain significance (VUS), likely benign (LB), or benign (B). Clinical phenotypes were annotated using Human Phenotype Ontology (HPO) terms and cross-referenced with genetic findings to establish genotype-phenotype correlations.

Sanger sequencing validation of SNVs: Specific primers were designed using Primer 5.0 (NCBI GenBank reference sequences). PCR amplification was performed with the KAPA2G Robust HotStart PCR Kit (KAPA Biosystems) in a 25 µL reaction volume on a Hema 9,600 thermal cycler (Zhuhai Hema). Amplified products were validated via 1.5% agarose gel electrophoresis and purified for bidirectional sequencing on an ABI 3730XL sequencer (Applied Biosystems). Sequence data were aligned to reference genomes using DNASTAR software (Lasergene v7.1).

qPCR validation of CNVs: Primers targeting candidate CNV regions, and the ALB reference gene were designed using Primer 5.0. qPCR reactions (20 µL) were prepared with KAPA SYBR^®^ FAST qPCR Kits (KAPA Biosystems) and run on a Roche LightCycler^®^ 480 (Roche Diagnostics). Amplification specificity was confirmed by melting curve analysis. Relative copy numbers were calculated using the 2^−ΔΔCt^ method, comparing Ct values of target and reference (ALB) genes. Experiments were conducted in triplicate, and statistical analysis was performed using GraphPad Prism v9.0.

## 3 Results

### 3.1 General characteristics

This study included eight pediatric patients (six males, two females) diagnosed with CSA due to *ERCC8* variants. All patients were initially misdiagnosed with cerebral palsy but were later confirmed to have CSA through genetic testing. Table 1 delineates the demographic and clinical attributes of the cohort. The mean age at symptom onset was 1.63 years (range: 6 months–2.5 years), and the average age at diagnosis was 3.75 years (range: 2–9.67 years). Five patients exhibited CS type I, two had CS type II, and one presented with CS type III. Unique perinatal histories were noted: Patient P4 (CS II) was conceived naturally despite maternal infertility and emotional distress during pregnancy; Patient P3 (CS II) had oligohydramnios and a knotted umbilical cord, requiring neonatal intensive care; and Patient P8 (CS I) showed intrauterine growth restriction and umbilical cord torsion. The mother of Patient P7 (CS III) was of advanced maternal age (39 years).

**TABLE 1:** Clinical characteristics of subjects with *ERCC8* gene variants.

Patients	P1	P2	P3	P4	P5	P6	P7	P8
Age of onset	2 years 6 months	2 years	6 months	8 months	1 year 2 months	2 years	2 years	2 years 2 months
Age at diagnosis	3 years	9 years 8 months	2 years	2 years 4 months	4 years 4 months	2 years 7 months	2 years 10 months	3 years 4 months
Sex	Female	Male	Male	Male	Male	Male	Male	Female
Consanguinity	-	-	-	-	-	-	-	-
Birth history	G1P1	G2P2	G1P1, oligohydramnios and umbilical cord knot	G1P1	G1P1	G3P2	G1P1	G1P1, intrauterine growth restriction and umbilical cord torsion
Birth weight	NA	4 kg	2.8 kg	3 kg	2.745 kg	2.8 kg	2.95 kg	2.75 kg
Initial diagnosis	Spastic diplegia	Spastic diplegia	Spastic quadriplegia	Spastic diplegia	Spastic quadriplegia	Spastic diplegia	Spastic diplegia	Spastic diplegia
Clinical Features – Typical Symptoms								
Growth disorder (as of last follow-up)	8 years 5 months: height 92 cm, weight 10 kg, both <−3SD	10 years: height 110 cm, weight 20 kg, both <−3SD	3 years: height 85 cm, weight 10 kg, both <−3SD	3 years 6 months: height 94 cm, weight 12 kg, both <−3SD	9 years: height 106 cm, weight 11 kg, both <−3SD	4 years 2 months: height 98 cm, weight 11.5 kg, both <−3SD	5 years 9 months: height 103 cm, weight 14 kg (<−2SD)	5 years: height 92 cm, weight 10.4 kg (<−3SD)
Malnutrition	+	+	+	+	+	+	-	+
Developmental delays	Started walking at 2 years, then regressed; currently 8 years and 6 months old unable to stand or walk independently	Head control at 4 months, independent sitting at 12 months, walking at 20 months, regressed after 3 years; first words at 12 months; currently 11 years old, requires assistive devices for standing	Developmental delay from infancy, independent sitting at 12 months; Unable to stand or walk independently at 3 years 6 moths(dead)	Head control at 3 months, rolling at 4 months, independent sitting at 7 months, crawling at 1 year; Around 2 years, could walk short distances indoors, currently 3 years and 5 months old unstable independent walking	Head control at 4 months, independent sitting at 12 months, first words at 2 years; Currently 9 years and 10 months old unable to stand or walk independently, requires orthotic assistance	Head control at 6 months; independent sitting at 12 months; first words at 18 months; Short periods of indoor walking since age 4, ataxic gait	Independent walking at 1 year 6 months; first words at 1+ year; Currently 5 years and 7 months old walks independently but unsteady, tiptoes, cannot climb stairs alone; hand tremors, instability, ataxia	Began supported walking at 1 year 7 months; independent walking at 2 years; Currently 4 years and 11 months old walks independently but unsteady, tiptoes; ataxia
Intellectual development	Severe	Severe	Severe	Moderate	Severe	Severe	Moderate	Moderate
Language	No words	No words	No words	Can call for people	Single words	No words	Can speak simple sentences	Can speak simple sentences
Facial dysmorphism	Deep-set eyes, enophthalmos, high-arched palate, micrognathia	Distinct facial features, prominent forehead, small earlobes, enophthalmos, progeroid appearance	Hypertelorism, micrognathia, progeroid appearance	Protruding ears, mild hypertelorism, micrognathia, no apparent progeroid features	Progeroid appearance, protruding ears, hooked nose, thin limbs, sunken eyes	Microcephaly, progeroid appearance, protruding ears, sunken eyes	Microcephaly, progeroid appearance	NA
Microcephaly	+	+	+	+	+	+	+	+
Brain MRI	Bilateral periventricular hyper intensities on T2, suggesting delayed myelination	Periventricular white matter abnormalities; bilateral basal ganglia changes; ventricular dilation; widened sulci and fissures; cerebellar atrophy	Bilateral periventricular white matter abnormal signals; Cisterna magna arachnoid cyst	Mild lateral ventricular enlargement, enlarged posterior fossa, cerebellar; Third and fourth ventricle dilation	Normal	Cisterna magna widening, arachnoid cyst possible, partial widening and deepening of sulci, cisterns, and fissures	Normal	Slightly widened left temporal extra-axial space
Seizures	-	-	-	-	-	-	-	-
EEG	NA	Normal	Abnormal	Normal	Normal	Normal	Normal	NA
Bilateral BAEP	NA	Abnormal	Abnormal	Abnormal	Normal	Abnormal	Normal	NA
Photosensitivity	-	+	-	+	+	+	+	-
Dental abnormalities	Delayed dentition, caries	Malocclusion, multiple caries	Delayed eruption, no caries	Caries, easily decayed teeth	Caries	None	Normal dentition, caries	Caries
Musculoskeletal abnormalities	Hip joint and lower limb osteoporosis; scoliosis; knee arthritis, pain, Achilles tendon contracture deformity	Scoliosis, pelvic tilt; Achilles tendon contracture deformity	Hip joint and lower limb osteoporosis, disuse bone changes; Achilles tendon and foot contracture	Achilles tendon contracture	Bilateral flat foot deformity	Hip joint and lower limb osteoporosis, bilateral knee valgus	Congenital genu valgum, Achilles tendon contracture, flatfoot	Tiptoeing gait, Achilles tendon contracture
Ocular abnormalities	NA	NA	+	NA	+, astigmatism	+, strabismus	+, astigmatism	-
Auditory abnormalities	-	ABR thresholds: left ear 95 dB nHL (no response), right ear 90 dB nHL	-	ABR thresholds: Left ear 70 dB nHL, right ear 50 dB nHL	-	-	ABR thresholds: Left ear 55 dB nHL, right ear 40 dB nHL	-
Clinical Features – Atypical Symptoms								
Persistent transaminase elevation (ALT/AST)	+	+	+	+	+	+	+	+
Other notable findings	Anhidrosis; thin limbs, large hands and feet; strabismus	Thin limbs, large hands and feet; strabismus; autistic traits; feeding difficulties	Recurrent respiratory infections, right cryptorchidism; cardiac ultrasound abnormalities	Short lingual frenulum	Thin limbs, left hydronephrosis; cryptorchidism	Cryptorchidism; cardiac ultrasound: atrial level fine bundle left-to-right shunt (2.5 mm)	-	Eczema; anhidrosis; hyperglycemia

F, female; M, male; NA, not available.

### 3.2 Clinical manifestations

**FIGURE 1:**
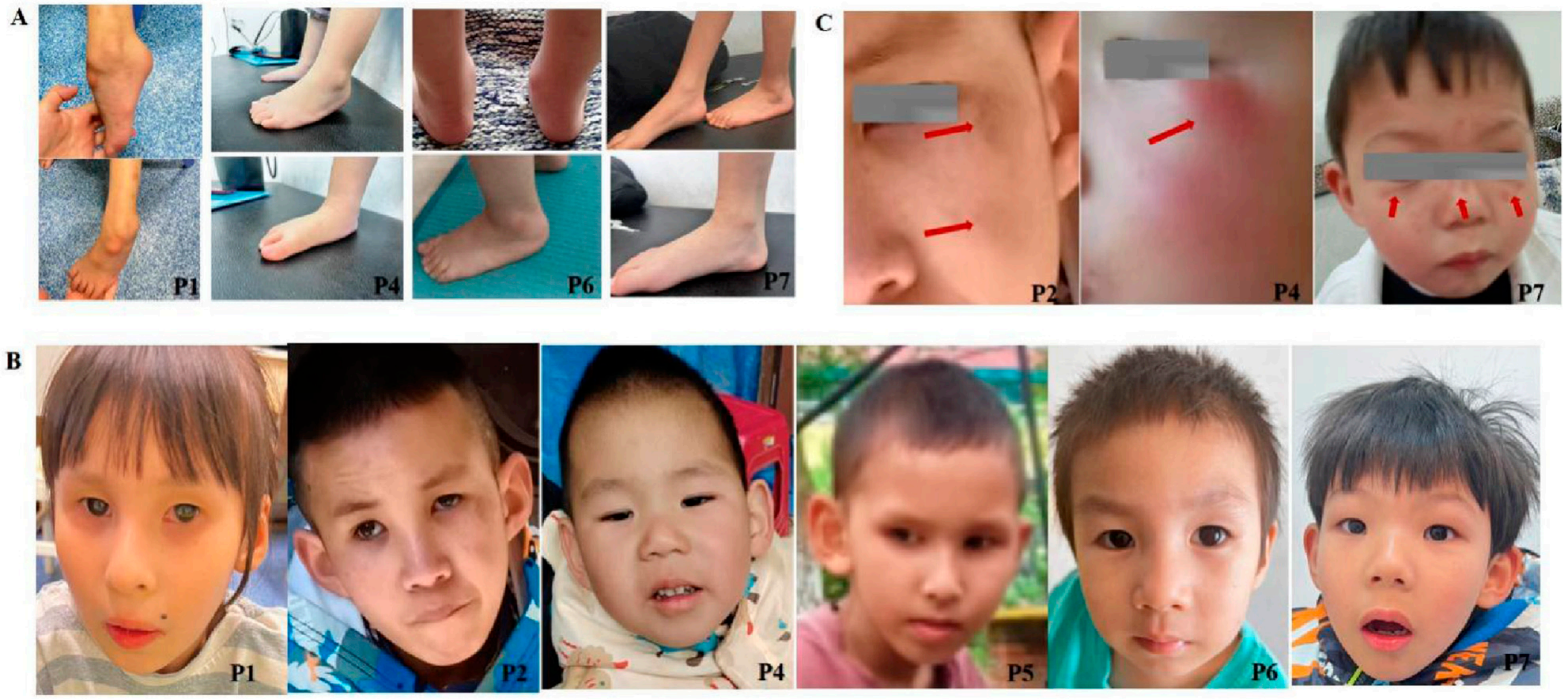
Clinical characteristics of CSA patients. **(A)** Achilles tendon contractures in CSA patients (P1, P4, P6, P7). Contractures were universally present, with severity influenced by disease subtype and rehabilitation status. P1 (8 years, no rehabilitation) exhibited severe contracture with ankle dorsiflexion <5°; P4 (3 years, ongoing rehabilitation) showed mild right (20°) and moderate left (10°) contractures; P6 (4 years, continued rehabilitation) displayed mild right (15°) and moderate left (5°) contractures; P7 (5 years, intermittent rehabilitation) presented mild left (20°) and moderate right (5°) contractures. **(B)** Progeroid facial features in CSA patients (P1, P2, P4, P5, P6, P7). Progressive aging-like changes, including micrognathia, protruding ears, and a high-arched palate, were observed, with overt progeroid features in severe cases. Patients’ ages: P1 (8 years), P2 (11 years), P4 (4 years), P5 (5 years), P6 (4 years), P7 (5 years). **(C)** Dermatological manifestations in CSA patients (P2, P4, P7). Photosensitivity-related skin changes, such as erythema, desquamation, and hyperpigmentation, were noted in sun-exposed regions. Anhidrosis was observed in two patients. P2 and P7 exhibited hyperpigmentation, while P4 showed erythema.

Global developmental delay was observed in all seven patients with CS I and CS II (7/7, 100%), while the CS III patient exhibited normal early development but progressive motor and cognitive regression from age three (1/1, 100%). Growth impairment and cachexia were prevalent (7/8, 87.5%) (Table 1). Milestone delays were universal, with five patients initially achieving independent ambulation before age two but later regressing. Currently, only two patients (P7 and P8) can walk independently, albeit with an unsteady gait (2/8, 25%). Achilles tendon contracture was present in all cases (Figure 1A).

Cognitive assessments revealed borderline intellectual function in two patients (2/8, 25%), mild cognitive impairment in one (1/8, 12.5%), and profound intellectual disability in five (5/8, 62.5%). Language abilities varied: two patients could form simple sentences (2/8, 25%), two could vocalize single words (2/8, 25%), and four were nonverbal (4/8, 50%)

**FIGURE 2:**
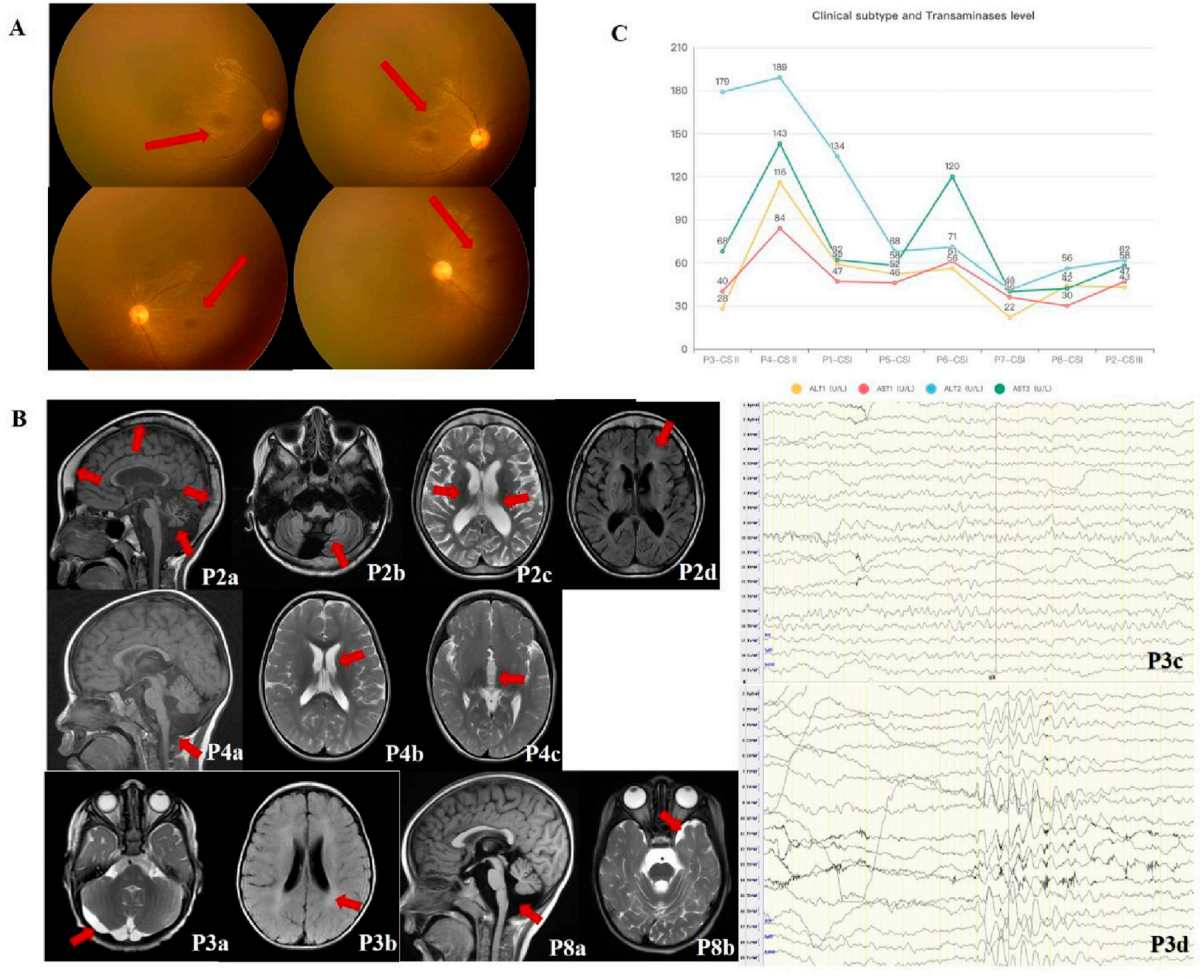
Imaging and laboratory findings in CSA patients. **(A)** Retinal abnormalities in CSA patients. Retcam fundoscopic images from P3 show fine retinal blood vessels at the posterior pole, poorly reflected macular light, and granular brownish pigmentation in the macular area. **(B)** Neuroimaging and EEG findings in CSA patients. Cranial MRI scans from P3 and P8 demonstrate white matter abnormalities (e.g., hypomyelination) and cerebellar atrophy. Key findings include: P2 (9 years 8 months): Cranial thickening, cerebellar atrophy (P2a, SAG T1WI), widened sulci (P2b, OAX T1WI), bilateral basal ganglia changes, ventricular dilation (P2c, OAX T2WI), and periventricular white matter abnormalities (P2d, OAX T2FLAIR). P3 (2 years): Delayed myelination (P3a, OAX T2WI) and an arachnoid cyst in the posterior fossa (P3b, OAX T2FLAIR). EEG showed increased background slow wave activity (P3c) and low-moderate amplitude θ rhythms in occipital regions (P3d). P4 (2 years): Enlarged posterior fossa, cerebellar hypoplasia (P4a, SAG T1WI), mild lateral ventricular enlargement (P4b, OAX T2WI) and ventricle dilation (P4c, OAX T2WI). P8 (3 years 4 months): Slightly enlarged cisterna magna (P8a, SAG T1WI) and widened left temporal extra-axial space (P8b, OAX T2WI). **(C)** Hepatic function analysis. Graphs depict elevated transaminase levels (ALT and AST) in CSA patients, with the highest levels observed in CS II patients (P3, P4), correlating with disease severity. Milder subtypes (CS I and CS III) exhibited comparatively modest hepatic dysfunction, suggesting a positive correlation between liver injury and disease severity.

All patients exhibited microcephaly (8/8, 100%). Six showed progressive progeroid facial changes (6/8, 75%), while two displayed overtly aged features early (2/8, 25%) (Figure 1B). Delayed dentition (7/8, 87.5%) and severe dental caries (6/8, 75%) were common. Photosensitivity-related skin changes (erythema, desquamation, hyperpigmentation) were noted in four patients (4/8, 50%), while two exhibited anhidrosis (2/8, 12.5%) (Figure 1C). Auditory abnormalities were documented in three cases (3/8, 37.5%) (Table 1). Visual evoked potential (VEP) abnormalities were found in two patients, and one exhibited macular degeneration (Figure 2A).

### 3.3 Neuroimaging and electrophysiological characteristics

Cranial MRI revealed white matter abnormalities in three patients (3/8, 37.5%), primarily in the anterior and posterior horns of the lateral ventricles and the posterior limb of the internal capsule. Cerebellar atrophy was identified in two cases (2/8, 25%) (Figure 2B). Additional findings included ventriculomegaly (1/8, 12.5%), extracerebral space enlargement (1/8, 12.5%), and partial widening of cerebral sulci and gyri (1/8, 12.5%). Two patients showed no abnormalities (2/8, 25%). Electrophysiological assessments in six patients revealed borderline abnormalities in one (1/6, 16.7%) and normal findings in five (5/6, 83.3%) (Figure 2B). Borderline EEG findings (P3) included θ rhythms (6–7 Hz) with diffuse 2.5–4 Hz slow waves and poor regulation, but did not meet definitive abnormality thresholds (e.g., slow waves >25% or epileptiform discharges). These suggest delayed brain maturation.

### 3.4 Hepatic function analysis

All patients exhibited elevated serum alanine aminotransferase (ALT) (normal: 7–30 U/L) and aspartate aminotransferase (AST) (normal: 14–44 U/L) levels (ALT: 28–116 U/L; AST: 30–189 U/L). Hepatic dysfunction correlated with disease severity, with the most pronounced abnormalities in the youngest and most severely affected CS II patient (P3). Milder subtypes (CS I and CS III) showed relatively lower ALT and AST levels. Progressive hepatic dysfunction was associated with disease progression, with CS II patients exhibiting the highest enzyme levels (Figure 2C). Table 2 details the *ERCC8* genetic variants, CS scoring, and hepatic function indices for all patients.

**TABLE 2:** *ERCC8* variants, CS scores, and hepatic function of patients.

Patients	P1	P2	P3	P4	P5	P6	P7	P8
CS Type	CS Ⅰ	CS Ⅲ	CS Ⅱ	CS Ⅱ	CS Ⅰ	CS Ⅰ	CS Ⅰ	CS Ⅰ
Variants	c.856A>T (p. Lys286) *, homozygous	Exon4 Deletion, homozygous	c.394_398del (p. Leu132Asnfs6); Exon4 deletion*, compound heterozygous	c.856A>T (p. Lys286); c.618-2A>G*, compound heterozygous	c.394_398del (p. Leu132Asnfs6); Exon4 deletion*, compound heterozygous	c.394_398del (p. Leu132Asnfs6) *, homozygous	Exon4 Deletion; Exon6-12 Deletion, compound heterozygous	c.394_398del (p. Leu132Asnfs6) *, homozygous
ALT (U/L)^a^	Diagnosed: 59 Last time: 134	Diagnosed: 43 Last time: 62	Diagnosed: 28 Last time: 179	Diagnosed: 116 Last time: 143	Diagnosed: 52 Last time: 68	Diagnosed: 56 Last time: 71	Diagnosed: 36 Last time: 41	Diagnosed: 44 Last time: 56
AST (U/L)^b^	Diagnosed: 47 Last time: 62	Diagnosed: 47 Last time: 58	Diagnosed: 40 Last time: 68	Diagnosed: 40 Last time: 68	Diagnosed: 46 Last time: 58	Diagnosed: 61 Last time: 120	Diagnosed: 36 Last time: 40	Diagnosed: 30 Last time: 42
Diagnostic Score(clinical and clinical-radiological)	12/20 19/39	15/20 23/39	6/20 11/39	15/20 21/39	16/20 27/39	10/20 14/39	9/20 17/39	6/20 9/39
Severity Score at Diagnosis	8/15	10/15	9/15	10/15	8/15	10/15	10/15	10/15
Current Severity Score	8/15	8/15	5/15	5/15	5/15	8/15	10/15	10/15
Achilles Tendon Contracture (Modified Ashworth Scale)	Diagnosed: 3 Last time: 4	Diagnosed: 2 Last time: 3	Diagnosed: 2 Last time: 1+	Diagnosed: 2 Last time: 2	Diagnosed: 3 Last time: 2	Diagnosed: 2 Last time: 1+	Diagnosed: 1+ Last time: 2	Diagnosed: 2 Last time: 1+

a and b: The time interval between two liver function tests ranges from 1 to 2 years.

### 3.5 Genetic analysis

**FIGURE 3:**
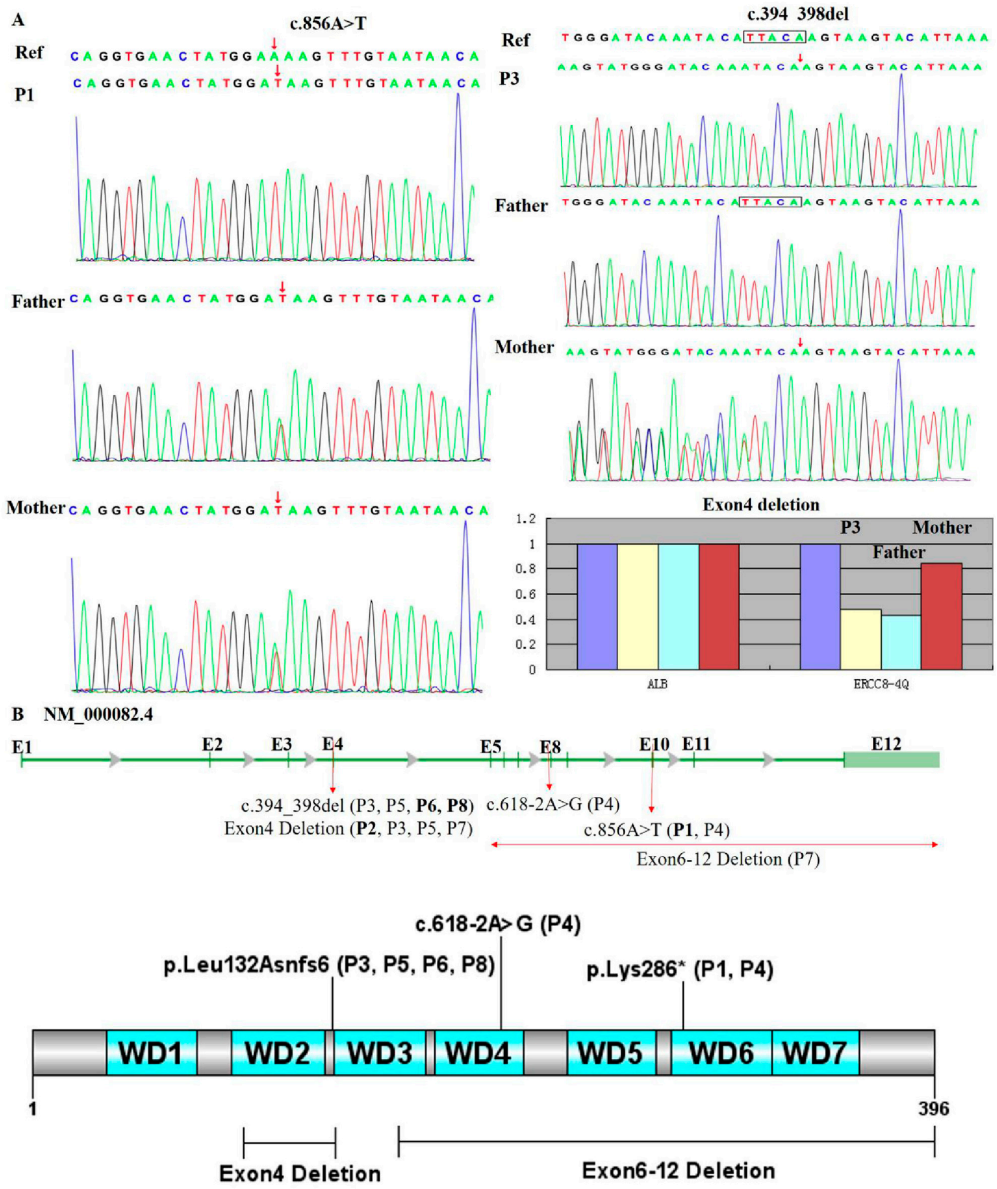
Genetic analysis of patient variants. **(A)** Sanger sequencing and qPCR confirmed that all patients carried pathogenic *ERCC8* variants, either in a homozygous or compound heterozygous state. Sequencing electropherograms for two individuals (P1, P3) are shown. P1 exhibits a homozygous c.856A>T variant (red arrow), while P3 shows compound heterozygous c.394_398del and Exon4 deletion. **(B)** Distribution of variants within the *ERCC8* gene (NM_000082.4) and its encoded CSA protein, highlighting the loss of critical functional domains leading to structural and functional impairment. The gene structure is depicted with exons (E1–E12) and mutation sites: c.394_398del (affecting P3, P5, P6, P8) in E4, exon 4 deletion (affecting P2, P3, P5, P7), c.618-2A>G (affecting P4) in E8, c.856A>T (affecting P1 and P4) in E10, and exon 6–12 deletion (affecting P7). The bar chart at the bottom illustrates the proportion of exon 4 and exon 6–12 deletions.

All patients harbored compound heterozygous or homozygous *ERCC8* variants (NM_000082.4), including exon deletions (Exon4, Exon6-12), a nonsense variant (c.856A>T, p. Lys286), a frameshift variant (c.394_398del, p. Leu132Asnfs6*), and a splice-site variant (c.618-2A>G). The genetic and clinical classifications were as follows: P1 (homozygous c.856A>T–CS I), P2 (homozygous Exon4 deletion–CS III), P3 and P5 (compound heterozygous c.394_398del and Exon4 deletion–CS II and CS I, respectively), P4 (compound heterozygous c.856A>T and c.618-2A>G–CS II), P6 and P8 (homozygous c.394_398del–CS I), and P7 (compound heterozygous Exon4 and Exon6-12 deletions–CS I). Parental analysis confirmed autosomal recessive inheritance (Figure 3A). No familial relationships were identified between patients sharing identical variants (P3/P5, P6/P8), suggesting independent occurrences. Notably, c.856A>T (rs1371445915) and c.618-2A>G were absent from the 1000 Genomes and gnomAD databases, indicating extreme rarity, while c.394_398del (rs774542633) had an allele frequency of 0.0001 in ExAC. The exon deletions disrupted the WD-repeat domain of the CSA protein: Exon4 deletion removed residues 93–133 (impairing WD2), and Exon6-12 deletion eliminated residues 161–396 (affecting WD3–7), leading to significant protein dysfunction (Figure 3B). All variants, inherited in trans with pathogenic or likely pathogenic alleles, were classified as pathogenic (P) per ACMG guidelines (PVS1, PM2_Supporting, PM3).

### 3.6 Rehabilitation therapy and follow-up

One CS II patient (P3) died from severe pneumonia and malnutrition at 3.5 years. The remaining seven patients exhibited malnutrition and growth retardation, with slow growth in height and weight after age three. Five patients (P3, P4, P5, P6, P8) who received rehabilitation therapy showed improvement in Achilles tendon contracture and gait stability, while three untreated patients (P1, P2, P5) experienced progressive symptom worsening. No pharmacological interventions for muscle tone reduction were administered.

## 4 Discussion

CS and CP share overlapping clinical features, particularly motor dysfunction and postural abnormalities, often leading to misdiagnosis. However, their etiologies, pathological mechanisms, and management strategies differ significantly. CP results from non-progressive central nervous system (CNS) injury occurring before, during, or shortly after birth (Graham et al., 2016). In contrast, CS is a progressive neurodegenerative disorder caused by pathogenic variants in *ERCC6* or *ERCC8*, leading to impaired DNA repair. The concept of the “CP mimic” emphasizes that certain genetic and metabolic disorders, including CS, can present with CP-like features, some of which exhibit neurodegeneration with variable postnatal onset (Pearson et al., 2019). Genetic testing is therefore indispensable for distinguishing atypical CP cases and identifying CS. This not only clarifies the underlying disorder but also prevents inappropriate interventions. A notable example from this study involved the mother of P8, who identified one of her twin fetuses as carrying the same *ERCC8* pathogenic variant as her affected child through prenatal genetic counseling and amniocentesis. This led to selective reduction and the birth of a healthy infant, underscoring the critical role of genetic screening in managing hereditary diseases. Patients presenting with movement abnormalities, joint contractures, and atypical posture—especially when accompanied by microcephaly or photosensitivity—should undergo genetic testing to exclude hereditary conditions. The diagnostic criteria for CS include progressive microcephaly, ophthalmic abnormalities (e.g., cataracts, retinal dystrophy), enophthalmos, delayed neurodevelopment, cognitive impairment, spastic-ataxic gait, atypical neuroimaging findings (e.g., combined white and gray matter involvement), peripheral demyelination, sensorineural hearing deficits, cutaneous photosensitivity, dental anomalies, and hepatic, renal, or vascular dysfunction. Collectively, these features differentiate CS from CP (Karikkineth et al., 2017; Stafki et al., 2023).

CS exhibits significant phenotypic heterogeneity, with distinct differences in onset and progression across subtypes. CS type II typically manifests within the first year of life, CS type I around 2 years of age, and CS type III later in childhood. Earlier onset generally correlates with increased disease severity (Spitz et al., 2021). In this study, growth parameters, including head circumference and stature, plateaued after age two, with most patients exhibiting moderate-to-severe malnutrition. Consistent with previous reports, CS type I cases initially followed normal growth trajectories but developed microcephaly by 2 months, with height and weight limitations evident between 5 and 22 months. In contrast, CS type II patients showed suboptimal birth parameters that progressively worsened (Baer et al., 2021). Caloric restriction has been shown to prolong lifespan in progeroid mouse models (Vermeij et al., 2016), suggesting that stringent weight regulation and avoidance of excessive nutrition may help decelerate premature aging in CS. Close monitoring of growth metrics, head circumference, and nutritional status, coupled with individualized dietary planning, is essential. Additionally, some CS patients exhibit anhidrosis, reflecting underdeveloped eccrine sweat glands, which aligns with known phenotypic characteristics (Landing et al., 1983). Comprehensive clinical evaluation, particularly of dermatological anomalies, is therefore pivotal for early diagnosis.

Hepatic dysfunction is a critical feature of CSA and should be integrated into its disease management framework. In this study, all patients exhibited elevated alanine aminotransferase (ALT) and aspartate aminotransferase (AST) levels, which correlated positively with disease severity and progressively worsened over time. This finding provides a quantifiable biochemical marker for assessing disease progression, although larger cohort studies are needed to validate these results while accounting for age-related variations and the natural history of the disorder. The hepatic abnormalities observed in CSA may stem from genomic instability and transcriptional stalling. Pathogenic variants in *ERCC6* or *ERCC8* impair transcription-coupled repair, causing RNA polymerase II to stall during elongation, leading to R-loop formation, exacerbating genome instability, and inducing hepatocellular injury (Apelt et al., 2021; Zhang et al., 2024). Beyond transaminase elevation, CSA patients may present with additional biochemical abnormalities. In this study, P3 exhibited pigmentary retinopathy, hyperammonemia, elevated lactate and lipid levels, and renal impairment, representing the earliest fatal case among the cohort. Six individuals demonstrated mild creatine kinase-MB (CK-MB) elevation, and two had congenital cardiac anomalies. Although previous reports have described hepatomegaly and sporadic cholestasis as predominant hepatic manifestations in CSA, no structural liver abnormalities were detected in our cohort (Komatsu et al., 2004). Endocrine dysfunction, including hyperinsulinemia and impaired glucose tolerance, was also observed, with consistently elevated glucose levels in repeated blood tests. Prior studies have documented diabetes onset in some adolescent CS type III cases (Ting et al., 2015), highlighting the need for comprehensive biochemical monitoring of hepatic, renal, cardiovascular, and endocrine parameters to enable holistic disease assessment and personalized management in CSA.

The HGMD Professional database (http://www.hgmd.cf.ac.uk/ac/gene.php?gene=ERCC8) has cataloged 96 pathogenic *ERCC8* variants, some of which may arise from founder effects. For instance, c.966C>A (p.Tyr322*) has an allele frequency of approximately 6.79% in Arab populations (Chebly et al., 2018), while c.551G>A has been proposed as a founder variant among Somali individuals (Laugel et al., 2010). Additionally, c.370_371del (p.L124Efs15) and c.484G>C (p.G162R) have been identified in Vietnamese patients (Duong et al., 2022), c.37G>T (p.Glu13*) in Indian cases (Narayanan et al., 2021), and c.1122G>C (p.Glu374Asp) and c.1053del in Iranian cohorts (Taghdiri et al., 2017; Mohammadi-Asl et al., 2018). In this study, all eight patients harbored compound heterozygous or homozygous *ERCC8* variants, including exon deletions, nonsense variants, frameshift alterations, and splice-site defects, all resulting in loss-of-function consequences. Notably, we report Exon6-12 Deletion for the first time, which disrupts the WD3-WD7 domains of CSA and is not yet cataloged in the HGMD or ClinVar databases. Our findings confirm the high carrier frequency of specific variants in the Chinese population, particularly Exon4 Deletion, c.394_398del, and c.856A>T, suggesting these variants are predominant pathogenic causes of CSA in this demographic. Recent research has linked c.856A>T (p. Lys286) * to hereditary neurological disorders in China (Zou et al., 2024). Haplotype analysis indicates that Exon4 Deletion and c.394_398del likely originated from founder variants within the Chinese population (Wang et al., 2017), reinforcing their role as major pathogenic variants. Additionally, cases of c.618-2A>G in combination with Exon4 Deletion have been reported in Chinese patients (Xie et al., 2017). Most individuals carrying *ERCC8* variants exhibit a CS type I phenotype (Laugel, 2013), consistent with our cohort, where 63% (5/8) of patients were classified as CS type I. Notably, P2, with a homozygous Exon4 Deletion, exhibited a CS type III phenotype, suggesting a possible association between this variant and a milder clinical course. P7, harboring compound heterozygous Exon4 Deletion and Exon6-12 Deletion, was categorized as CS type I, indicating that Exon6-12 Deletion may exacerbate the phenotype to a limited extent. P1, with homozygous c.856A>T, was classified as CS type I, whereas P4, carrying c.856A>T and c.618-2A>G, displayed a CS type II phenotype, suggesting that c.618-2A>G may contribute to increased disease severity. Phenotypic differences between P3 and P5, both carrying c.394_398del and Exon4 Deletion, highlight the potential influence of genetic background or modifier genes on disease classification. Despite apparent genotype-phenotype correlations, interindividual variability suggests that environmental factors, modifier genes, and broader genetic backgrounds significantly impact disease expression. For instance, maternal health during pregnancy may modulate phenotypic severity. This study expands the known mutational spectrum of *ERCC8* and underscores the critical role of genetic analysis in confirming diagnoses, elucidating disease mechanisms, and guiding precision medicine approaches. Future studies integrating functional assays and multicenter cohorts are warranted to further dissect the genetic determinants underlying CSA’s phenotypic heterogeneity and its modifying factors.

Although the pathogenesis of CSA remains incompletely understood and no definitive treatments exist, the clinical subtype provides insight into disease progression and prognosis. The expected lifespan for CS type II patients is approximately 5–6 years, while CS type I individuals typically survive until around 16 years of age, and CS type III cases may reach 30 years. Mortality is predominantly attributed to respiratory failure, with some patients succumbing to renal failure. Current management strategies remain largely symptomatic, encompassing specialized education, structured rehabilitation programs, meticulous nutritional planning, and protective measures against sun exposure to safeguard the skin and retina. Routine ophthalmologic, otolaryngologic, and dental evaluations are recommended, alongside regular monitoring of hepatic and renal function, blood pressure, and glucose levels. Pharmacologic interventions, including antiepileptic and antispasmodic agents, may be warranted in select cases. Metronidazole must be strictly avoided due to severe hepatotoxicity risks, as evidenced by multiple cases of acute liver failure and death in CS patients (Ataee et al., 2020; Hunaut et al., 2022). A natural history study identified eight cases of metronidazole-induced liver failure in CS patients (8% of the cohort), with three fatalities occurring within 6–11 days of administration, reinforcing its absolute contraindication (Wilson et al., 2015). Additionally, Achilles tendon contracture is a common complication in CSA, arising from prolonged muscle hypertonia and motor dysfunction, which progressively restrict joint mobility and exacerbate disability. Our findings suggest that structured rehabilitation significantly enhances motor function, particularly when initiated in early disease stages, delaying the onset of contractures and improving overall quality of life. Effective rehabilitation modalities include static stretching (SS) and proprioceptive neuromuscular facilitation (PNF) stretching, which alleviate spasticity by increasing joint range of motion and elongating musculotendinous units (Kruse et al., 2022). Additionally, ankle-foot orthoses (AFOs) help maintain physiological joint alignment, enhance postural stability, and mitigate spasticity and deformities (Firouzeh et al., 2021). Thus, tailored rehabilitation and strict avoidance of metronidazole are critical in managing CS to enhance quality of life and reduce mortality risks.

## 5 Conclusion

CS presents with a broad clinical spectrum, with substantial differences in onset, progression, and prognosis across subtypes, complicating diagnosis. Early genetic testing is crucial for definitive diagnosis, facilitating genetic counseling, and enabling prenatal screening to improve reproductive decision-making within affected families. Routine hepatic function assessment should be incorporated into long-term follow-up protocols, given its relevance in disease monitoring. Furthermore, early and sustained rehabilitation interventions significantly improve motor function and overall quality of life. This study provides novel insights into CSA diagnosis and treatment, highlighting the necessity of a multidisciplinary approach in the management of this rare disorder.

## Data Availability

The ERCC8 variants NM_000082.4: (c.856A>T, p. Lys286), c.394_398del, p. Leu132Asnfs6*), and c.618-2A>G were submitted to the LOVD database, with the LOVD Variant ID: https://databases.lovd.nl/shared/variants/0001045051#00000962, https://databases.lovd.nl/shared/variants/0001045052#00000962, https://databases.lovd.nl/shared/variants/0001045053
